# Reply to Chandra: Severe Acute Respiratory Syndrome-Coronavirus-2 variant analysis reveals within-host selection of intra-host single nucleotide variants

**DOI:** 10.1073/pnas.2206919119

**Published:** 2022-08-01

**Authors:** Laura Bashor, Roderick B. Gagne, Angela Bosco-Lauth, Richard A. Bowen, Mark Stenglein, Sue VandeWoude

**Affiliations:** ^a^Department of Microbiology, Immunology, and Pathology, Colorado State University, Fort Collins, CO 80523;; ^b^Department of Pathobiology, Wildlife Futures Program, University of Pennsylvania School of Veterinary Medicine, Kennett Square, PA 19348;; ^c^Department of Biomedical Sciences, Colorado State University, Fort Collins, CO 80523

We thank the author (1) for the response to our publication (2) reporting SARS-Coronavirus-2 (SARS-CoV-2) within-host variants in four experimentally infected mammalian species (dog, cat, hamster, ferret) and variants that emerged during viral stock expansion. Although we agree with some of the statements presented, we feel that we addressed the most critical points in our original manuscript. We believe that the conclusions we made are well supported by our analyses and, thus, disagree with the statement that the manuscript requires “significant modification” (1). Furthermore, there were a number of inaccuracies in the letter as explained below.

We agree that the viral titer of the original sample is an important consideration in this type of study. However, we did not “claim that viral titer does not matter.” We noted that “[v]iral titer did not seem to determine the observed variant richness.” This sentence refers to figure 3A of ref. 2, which shows that within-host variants—detected in both technical replicates above 3% allele frequency—did not vary as an obvious function of sample titer. This sentence is well supported by the data, and the verb “seem” is appropriately understated. The author (1) conflated other quantities with viral titer, including total sequencing reads and viral copy number. Although qRT-PCR can provide important context, we did not measure viral copy number in this study.

Chandra (1) noted that “[c]orrect data analysis” is “crucial for the calculation of transmission bottlenecks.” Bottlenecks are an interesting aspect of viral evolution but were not evaluated in our reported work. The author (1) also states that a “large number of (random) false iSNVs [have been] included.” Although intra-host single nucleotide variants (iSNVs) may occur randomly, we strongly disagree that the majority of variants reported that were detected at >3% frequency in two technical replicates were false. This conclusion is supported by other empirical studies (3).

We agree that “there needs to be a certain minimum frequency threshold of, say, 3% for identifying iSNVs even at very high viral titers” (1). In fact, in our methods, results, and figure legends, we clearly stated that we used a cutoff of 3% prevalence for our primary analyses. Following the identification of emergent variants in animals above the 3% threshold and at >50% in one individual or present in all individuals of a species, we assessed the frequency of specific variants detected in animals in the viral stock used for inoculation. We hypothesized that inoculum variants present at 0.1 to 3% that subsequently increased in frequency in animal-derived samples may have been under positive selection within the new host. There is good precedent for this type of observation (4). We did not state, as is implied by the author, that all 546 of the very low–frequency variants were “real.”

In conclusion, we appreciate this active engagement with our work and the opportunity to respond to the concerns of the commentator. We believe that our manuscript clearly and repeatedly states the limitations of the sequencing and analysis approaches used, and the issues raised do not invalidate our primary conclusions.
